# A Computational Study on the Role of Gap Junctions and Rod I*_h_* Conductance in the Enhancement of the Dynamic Range of the Retina

**DOI:** 10.1371/journal.pone.0006970

**Published:** 2009-09-24

**Authors:** Rodrigo Publio, Rodrigo F. Oliveira, Antonio C. Roque

**Affiliations:** 1 Computational Neuroscience Unit, Okinawa Institute of Science and Technology, Okinawa, Japan; 2 Computational and Experimental Neuroplasticity Laboratory, The Krasnow Institute for Advanced Study, George Mason University, Fairfax, Virginia, United States of America; 3 Department of Physics and Mathematics, FFCLRP, University of São Paulo, Ribeirão Preto, Brazil; Lund University, Sweden

## Abstract

Recent works suggest that one of the roles of gap junctions in sensory systems is to enhance their dynamic range by avoiding early saturation in the first processing stages. In this work, we use a minimal conductance-based model of the ON rod pathways in the vertebrate retina to study the effects of electrical synaptic coupling via gap junctions among rods and among AII amacrine cells on the dynamic range of the retina. The model is also used to study the effects of the maximum conductance of rod hyperpolarization activated current I*_h_* on the dynamic range of the retina, allowing a study of the interrelations between this intrinsic membrane parameter with those two retina connectivity characteristics. Our results show that for realistic values of I*_h_* conductance the dynamic range is enhanced by rod-rod coupling, and that AII-AII coupling is less relevant to dynamic range amplification in comparison with receptor coupling. Furthermore, a plot of the retina output response *versus* input intensity for the optimal parameter configuration is well fitted by a power law with exponent 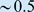. The results are consistent with predictions of more theoretical works and suggest that the earliest expression of gap junctions along the rod pathways, together with appropriate values of rod I*_h_* conductance, has the highest impact on vertebrate retina dynamic range enhancement.

## Introduction

One of the mechanisms used by the retina to operate over a wide range of brightness conditions is signal segregation into distinct pathways, all of which converge to the output layer of ganglion cells. In the mammalian retina, two different rod pathways are responsible for carrying rod signals to on-center (ON) ganglion cells [1]–[3]. In the primary ON rod pathway, rods make electrical synapses via gap junctions with neighboring rods and sign inverting chemical synapses with rod bipolar cells. These latter make excitatory chemical synapses with AII amacrine cells, which are electrically coupled via gap junctions among themselves and with ON cone bipolar cells [4], [5]. Finally, cone bipolar cells transmit the rod signals to ON ganglion cells via excitatory chemical synapses. In the secondary ON rod pathway, rods transmit signals to cones via electrical synapses. Subsequently, cones make sign inverting chemical synapses with ON cone bipolar cells, which relay the rod signals to ON ganglion cells via excitatory chemical synapses. The existence of electrical synapses mediated by gap junctions in these circuits suggests an important role for electrical coupling in the rod light processing pathways in the retina [2], [3].

Experimental and modeling works on photoreceptors of vertebrate species have shown that electrical coupling among rods improves the signal-to-noise ratio and extends the dynamic range of the rod output to rod bipolar cells [6], [7]. Electrical coupling in a network of simulated AII amacrine cells also improves the signal-to-noise ratio of this network [8]. Lack of electrical coupling between AII amacrine cells and a population of ON cone bipolar cells may affect the sensitivity of scotopic vision [9].

Theoretical works on a sensory layer of electrically coupled excitable elements like e.g. the photoreceptor layer in the retina have shown that coupling enhances the dynamic range of the layer and the response of this system is well-fitted by a power law, where the output is proportional to a power (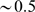) of its input [10]–[13]. However, since these works did not address the problem of a multi-layered and not random network like the retina the predictions that can be drawn from it, though insightful, are limited.

In this paper, we used a minimal conductance-based model of the two ON rod processing pathways of the vertebrate retina to investigate the effects of cell coupling via gap junctions with different connectivity degrees on the dynamic range of the retina. In particular, we studied the effects of variable connectivity degrees among two different cell populations of the retina, namely rods and AII amacrine cells. In our model, each cell population is represented by a two-dimensional square array and the connectivity degree of each layer is defined as the number of connections, on the average, that each cell makes with its first neighbors.

We used conductance-based neuronal models because they allow the investigation of the interacting effects of network connectivity degree and cellular properties on the dynamic range of the retina. The rod hyperpolarization activated current I*_h_* has been shown to increase the operational range of the single rod [14]–[16]. Therefore, we ask how these two properties acting simultaneously but at different levels will affect the dynamic range of the model.

We studied the effects of combinations of different connectivity degrees of rods (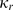) and AII amacrine cells (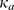) with different maximum I*_h_* conductances 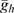 on the dynamic range of the retina. The simulation results show that gap junctional coupling among rods increases the dynamic range of the retina in comparison with the uncoupled cases. For realistic values of 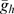 [15], [17], this increase is maximal for a small non-zero value of the rod-rod connectivity degree. The results also show that coupling among AII amacrine cells has little effect on the dynamic range of the retina.

## Methods

### Compartmental models

The retina model is made of conductance-based models of the following cells: rods, cones, rod and cone bipolar cells, AII amacrine cells and a ganglion cell. These models were adapted from previously published models. The values of passive properties and ionic current parameters for these cell models, which were modified by us for this work, are given in Tables 1 and 2 respectively. The values of other parameters and the equations describing the dynamics of their ionic channels, which were kept as in the original models, are given in the Supporting Information Text S1 and Supporting Information Tables S1–S5.

**Table 1:** Passive parameters of the neuron models. C*_m_* is the membrane capacitance and C*_rest_* is the membrane resting potential.

	Diameter	Lenght	C*_m_*	V*_rest_*
Rod	8 (µm)	8 (µm)	20 (pF)	−38 (mV)
Cone	8 (µm)	8 (µm)	20 (pF)	−42 (mV)
Bipolar cell	8 (µm)	8 (µm)	10 (pF)	−38 (mV)
AII amacrine cell	7 (µm)	7 (µm)	20 (pF)	−69(mV)
Ganglion cell	25 (µm)	25 (µm)	20 (pF)	−65 (mV)

**Table 2:** Ionic channels parameters of the neuron models. 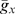 is the maximum conductance in nS for a specific ionic channel and E_x_ is the reversal potential in mV for this channel.

	Rod	Cone	Bipolar cell	AII amacrine cell	Ganglion cell
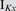	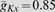	—-	—-	—-	—-
	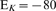	—-	—-	—-	—-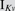	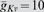	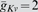	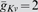	—-	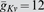
	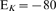	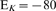	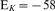	—-	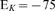
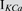	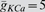	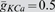	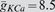	—-	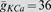
	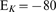	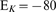	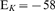	—-	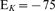
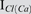	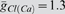	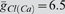	—-	—-	—-
	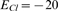	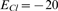	—-	—-	—-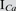	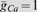	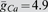	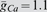	—-	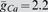
	—-	—-	—-	—-	—-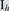	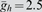	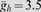	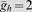	—-	—-
	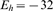	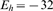	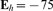	—-	—-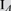	—-	—-	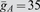	—-	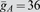
	—-	—-	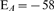	—-	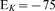
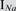	—-	—-	—-	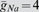	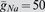
	—-	—-	—-	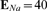	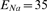
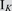	—-	—-	—-	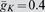	—-
	—-	—-	—-	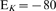	—-

**Figure 1:**
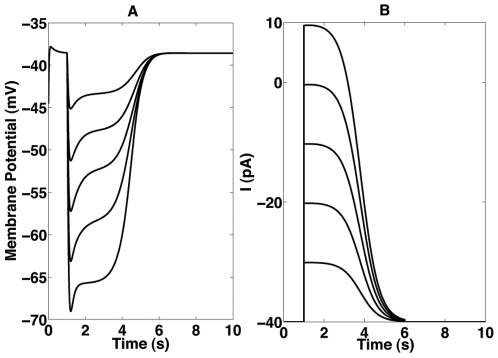
Simulated photocurrents used as rod input (B) and the respective rod responses (A). (A) Rod responses to the five simulated photocurrents. From top to bottom, traces correspond to photocurrents from 10 pA to 50 pA. (B) Photocurrent time course for five different photocurrent amplitudes, from bottom to top: 10 pA, 20 pA, 30 pA, 40 pA and 50 pA.

The rod model is a modified version of a single compartment model described by us elsewhere [16]. The modified model has six ionic currents (I*_Kx_*, I*_h_*, I*_Kv_*, I*_KCa_*, I*_Ca_*, and I*_Cl(Ca)_*). Instead of simulating the transduction process, the rod model uses a simulated photocurrent waveform as input. It is given by the expression [17]:
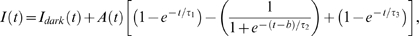
(1)where 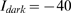 pA represents the dark current, 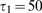 ms, 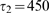 ms, 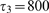 ms and 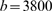 ms are constants, and 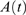 is a step function that represent the photocurrent amplitude. Figure 1(B) shows the photocurrent time course for five different amplitude values.

The photocurrent is injected in the rod compartment to simulate changes in the dark current caused by the light transduction process. The rod model responses to the five photocurrents waveforms are given in Figure 1(A). They are very similar to the ones observed experimentaly, exhibiting a hyperpolarized response peak followed by a plateau at a slightly less negative potential value [14], [18]. At the end of stimulus the rod membrane potential returns to its resting value of −40 mV.

The cone model is a modified version of the single compartment model of [17] with five ionic currents (I*_h_*, I*_Kv_*, I*_KCa_*, I*_Ca_*, and I*_Cl(Ca)_*). with similar dynamics as the rod model and parameters described in Table 2. Since the model simulates only the rod-mediated circuits, each cone receives only the dark current I*_dark_* as input instead of receiving the photocurrent waveform described above.

The rod bipolar cell model is a modified version of the single compartment model of [19]. It has five ionic currents (I*_Kv_*, I*_h_*, I*_A_*, I*_KCa_*, I*_Ca_*,) and their active properties were kept as in the original work of Usui *et al.*. We modified the compartment dimensions and adjusted its membrane capacitance so that the model voltage response to injected current correctly reproduces experimentally responses [20]–[22], exhibiting sustained depolarization during stimulus presentation followed by fast return to resting state after stimulus removal (data not shown).

The cone bipolar cell model is entirely similar to the rod bipolar cell model, but without the I*_A_* current [19].

The third neuron in the rod pathway is the AII amacrine cell. Evidences show that most of these cells have only sodium and potassium voltage-gated channels, and can produce spikes under specific *in vitro* circumstances [23], [24]. We modeled this cell as a single compartment with only sodium and potassium channels with parameters and dynamics taken from other works [23], [24]. The model is able to reproduce the single spike characteristic of this cell followed by a sustained hyperpolarization during stimulus application [24].

For the ganglion cell we used the single compartment model of Fohlmeister and Miller [25]. It was built from experimental data and is able to respond in a wide frequency range, from less than one to more than hundred spikes per second [25]. Taking advantage of the ganglion cell function in the circuitry: *i)* a natural integrator of signals from upstream processing layers; *ii)* the main output channel of the vertebrate retina and its natural wide response range, we use its activity to measure the dynamic range of the whole system.

### Synaptic connections and network topology

All electrical synapses in the model were modeled as a single resistance connecting two neighboring cells [6], [26].

The chemical synapses between rods and rod bipolar cells, rod bipolar cells and AII amacrine cells, and cone bipolar cells and ganglion cells are glutamatergic ribbon synapses. They are graded synapses specialized to continuously release glutamate as the stimulus intensity changes [27], [28]. A detailed model of ribbon synapse has been proposed by Sikora *et al.* [29] but instead of using it, which would increase the computational cost of our simulation, we used a simplified model of graded synapse [30] with parameters adapted from the model of Sikora *et al.*. This model is described below.

Our adapted chemical synaptic model simulates both the AMPA and the mGluR6 glutamate receptors present in the primary and secondary retina rod pathways. AMPA receptors are found in AII amacrine and ganglion cells [31], [32] and metabotropic mGluR6 receptors are found in bipolar cells [33]. These two receptor types were modeled by the same equations [30]. The equation for the synaptic current that is injected in the postsynaptic neuron is given by:
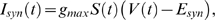
(2)where 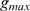 is the maximum conductance and 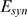 is the reversal potential. The variable 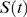 determines the activation level of the synapse. Its time variation as a function of the presynaptic cell voltage is given by:
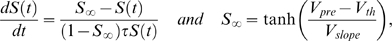
(3)where 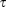 is a time constant, V*_pre_* is the presynaptic membrane potential and V*_th_* is the voltage threshold to activate the synapse. The values of the parameters of these equations are given in Table 3.

**Table 3:** Parameters of the graded chemical synapse model.

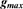	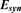	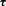	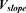
2.56 (nS)	0 (mV)	10 (ms)	10 (mV)

**Figure 2:**
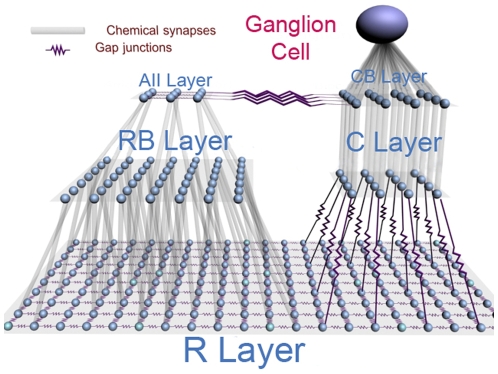
A scheme of the retina network model. Rods in the R layer are connected by electrical synapses both among themselves and with cones in the C layer, and by chemical synapses with rod bipolar cells in the RB layer. Rod bipolar cells in the RB layer are connected by chemical synapses with AII amacrine cells in the AII layer. AII amacrine cells in the AII layer are connected among themselves by electrical synapses and with cone bipolar cells in the CB layer. Cone bipolar cells in the CB layer receive chemical synapses from cones in the C layer and send chemical synapses to the ganglion cell.

The retina network model consists of a set of two dimensional rectangular grids representing a small area of the retina (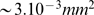) [1], on which model neurons are arranged. A scheme of it is shown in Figure 2. The photoreceptor layer contains 1500 rods (R) arranged on a 30×50 grid and 16 cones (C) arranged on a 4×4 grid; the layer of rod bipolar cells (RB) has 100 cells arranged on a 10×10 grid; the layer of cone bipolar cells (CB) contains 4 cells arranged on a 2×2 grid; the layer of AII amacrine cells (AII) has 9 cells arranged on a 3×3 grid; and the final layer, which is the retina output layer, consists of only one ganglion cell.

The number of cells on each layer was chosen to preserve, approximately, the convergence factors of each cell type to a ganglion cell for a region of the cat retina located at about 0.4–0.5 mm from the area centralis (which corresponds to the fovea in primates) [1]. This corresponds to an excentricity of 2 degrees [1]. Although very close to area centralis, the density of rods in this region is much higher than the density of cones [34]. The pattern of electrical and chemical synaptic connections in the retina model was determined as follows.

Each rod in the R layer makes an electrical synapse with each one of its first neighbors with probability 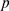, which can have one of the values: 0, 0.25, 0.5, 0.75 or 1. The first and the last cases are deterministic and correspond to situations in which rods are, respectively, uncoupled or completely coupled with their first neighbors. The other three cases correspond to situations in which rods are coupled, on average, with one, two or three of their first neighbors respectively. These five cases will be represented here by a connectivity index 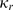 = 0, 1, 2, 3 and 4. All gap junctions between rods have the same conductance value of 0.5 nS [26].

For the electrical synapses between cones and rods, we considered that each cone in the C layer is coupled by gap junctions with 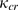 randomly chosen rods in the R layer, where 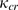 has the same value of the connectivity index 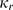 used for rod-rod coupling. All electrical synapses between cones and rods have the same conductance value of 0.2 nS [35].

Electrical coupling among cells in the AII layer was determined in the same fashion as for the R layer. The average number of connections between each AII amacrine cell and its first neighbors was given by a connectivity index 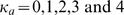, not necessarily equal to the one of the R layer. The conductances of these electrical synapses were are all fixed at 0.2 nS [24].

For the electrical synapses between AII amacrine cells and cone bipolar cells, we considered that each AII amacrine cell is electrically coupled with two randomly chosen cells from the CB layer. The conductances of these electrical synapses also had their values fixed at 0.2 nS [24].

According to the topology of each grid, cells belonging to the borders of the grids make a number of electrical synapses equal to 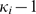, with 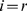 or 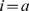.

The pattern of connections by chemical synapses was determined from the divergence factors between cell layers given by Sterling *et al.* [1]. We considered that: (1) each rod in the R layer is connected with two randomly chosen cells in the RB layer; (2) each cone in the C layer is connected with one randomly chosen cell in the CB layer; (3) each rod bipolar cell in the RB layer is connected with three randomly chosen cells in the AII layer; and (4) all four cone bipolar cells are connected with the ganglion cell.

For each simulation we assumed that a light flash of a given strength was presented to the entire R layer. However, to account for the fact that photon absorption is probabilistic [36] the number of activated rods at each experiment is a random number from an uniform distribution centered at 35 percent of the total with width of 10 percent. The actual number and the identity of rods which produce responses varied randomly within this fixed range from simulation to simulation, independently of flash strength. This assumption was made because our model simulates a very small area of the retina, so that one can assume that the fraction of activated rods is approximately constant for flashes of different intensities. In each simulation we considered that all rods that responded to a flash generated a photocurrent with the same amplitude and duration. Five different flash intensities were considered, corresponding to the five photocurrents shown in Figure 1(B) with amplitudes varying from 10 pA to 50 pA in steps of 10 pA and 5 s of duration.

To obtain the response of the ganglion cell for each flash intensity, we simulated the application of the corresponding photocurrent to the R layer for a period of 5 seconds as described in the previous paragraph. The firing frequency was calculated by counting the number of spikes generated by the ganglion cell during this 5-s period and dividing it by this period. We consider 5 seconds as a period suficiently long for a reliable estimation of ganglion cell firing frequency with a single realization of each experiment.

**Figure 3:**
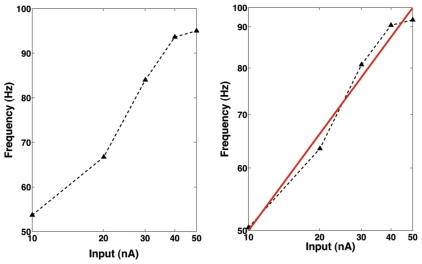
Firing frequency of the ganglion cell as a function of the photocurrent amplitudes used as stimuli for the case with 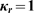 and 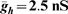. (A) Linear-log plot. The dots correspond to the experimental measures and the straight lines were included only to guide the eyes. (B) Log-log plot. The gray line shows the best-fit power law curve with exponent 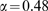. The root mean square error (RMSE) for the fit is 1.698 Hz.

The dynamic range 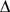 of the ganglion cell (or retina, because in our model they are the same) was calculated as [11]:
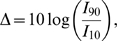
(4)where I_90_ and I_10_ are, respectively, the stimuli intensities for which the firing frequencies of the ganglion cell are 10 percent below the maximum and 10 percent above the minimum (Figure 3A).

The simulations were performed in NEURON 6.0 [37], [38] and the numerical integration of the equations was performed using the backward Euler method with a time step of 0.01 ms.

## Results

The retina model was used to study the effects of 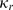, 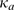 and 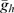 of the rod on the dynamic range of the retina. Our study consisted of two different experiments. In the first one, 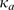 was fixed at a given value and we measured the dynamic range of the retina for all possible combinations of the five 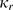 values with six different values of 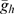. The six 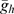 values vary in the range from from zero (blocked channel) to 2.5 nS in steps of 0.5 nS [15], [17]. In the second experiment, we fixed 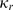 and measured the dynamic range of the retina for all possible combinations of the five 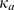 values with the six 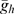 values.

In our first experiment, we fixed 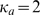. Then, we determined the dynamic range of the retina for all possible combinations of the values of 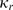 and 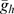 described in the previous section. The response curve of the ganglion cell for one of these combinations, namely 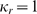 and 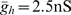 is shown in Figure 3. Each point in Figure 3 gives the firing frequency of the ganglion cell for the corresponding value of the photocurrent amplitude calculated as described in the previous section. This *f x I* curve can be well approximated by a power law 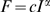, where F is the ganglion cell firing frequency, I is the photocurrent amplitude, 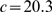 and 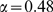. This result is compatible with available experimental data [2], [3] and is in good agreement with predictions from more abstract toy models [10]–[13].

**Figure 4:**
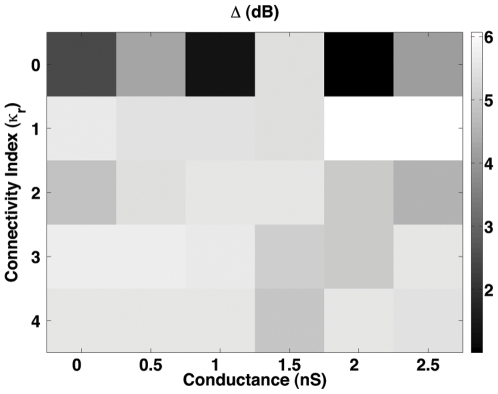
Dynamic range of the retina model for different combinations of 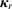 and 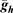 of the rod. In this case, 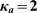. A gray scale was used to indicate the dynamic range 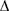 in decibels.

The dynamic ranges calculated for all combinations of 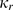 and 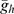 are shown in Figure 4. The dynamic range values shown in Figure 4 are low, always below 6 dB. This is a consequence of the fact that the range of photocurrent amplitudes used by us (10 pA–50 pA) does not correspond to the entire scotopic range. The superior limit (50 pA) corresponds to the approximate saturation value for scotopic vision [39] but the inferior limit (10 pA) is above the sensitivity threshold, which can be as low as 1 pA for a single photon detection [14]. Therefore, the results presented here should be viewed as referring to the dynamic range-enhancing effects of gap junctional coupling only for the restricted range of stimuli considered.

The dynamic range maxima in the diagram of Figure 4 occur for 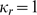 and the high conductance values 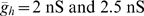. These maxima are the only values in the diagram which are above 6 dB. The response curve of the ganglion cell for one of these optimal pair of parameters, namely 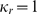 and 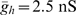, is shown in Figure 3. The fact that the maximal dynamic range in our model corresponds to a power law response curve may be related with suggestions from theoretical models and experimental evidence [13], [40]–[44] that optimal processing properties of sensory and neural systems in general are attaimed at or near a critical point of a phase transition.

**Figure 5:**
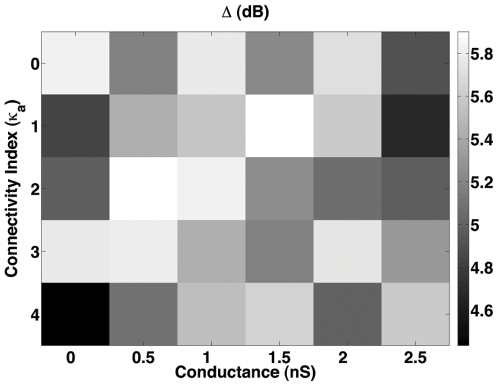
Dynamic range of the retina model for different combinations of 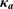 and 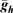. In this case, 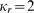. The gray scale gives the dynamic range 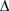 in decibels.

The second experiment was performed to assess the effect of the connectivity index of AII amacrine cells on the dynamic range of the retina. In this case, we fixed 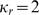 and tested all possible combinations of 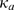 with 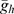 of the rod. The main objective of this experiment was to investigate if electrical coupling among AII amacrine cells would improve the dynamic range for one of the coupled rods cases. The results of this experiment are shown in Figure 5.

Figure 5 shows that, for 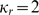, electrical coupling among AII amacrine cells has little effect on the dynamic range of the retina. For all combinations of 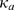 and 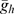 tested the dynamic range always remained within a narrow range between 4.5 e 5.9 dB, which is below the dynamic range maxima obtained for 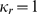. This result suggests that alterations in the electrical connectivity index of AII amacrine cells are not capable of significantly improving the dynamic range of the retina beyond what had already been attained by gap junctional coupling among rods.

**Figure 6:**
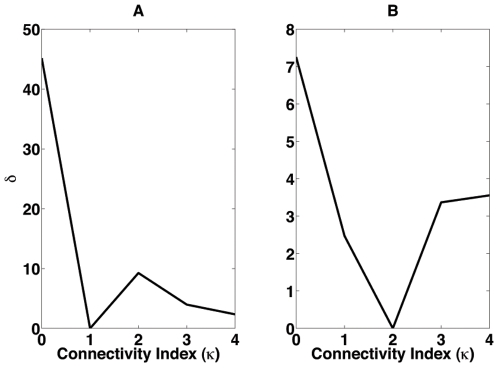
Line-averaged dynamic range percent variation in relation to its maximum as a function of the connectivity index for the R layer (A) and the AII layer (B).

To quantify the dynamic range variability as a function of the connectivity index for a given layer, we define the line-averaged dynamic range percent variation in relation to the maximum line-averaged value (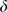) as:
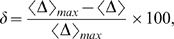
(5)where 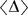 is the average dynamic range over 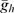 calculated for each 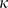 value and 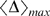 is the maximum value of 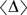 for a diagram. The behavior of 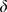 for the two experiments described here is shown in Figure 6.

The use of 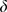 allows a study of the effect of the connectivity index of a given layer, 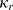 or 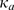, on the dynamic range of the retina independently of the value of 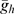. It shows that variations in the connectivity index of rods have a much larger effect on the dynamic range of the retina than variations in the connectivity index of AII amacrine cells. Figure 6A shows that electrical coupling among rods produces increases in the line-averaged dynamic range of the order 30–40 percent in comparison with the uncoupled rods case. On the other hand, Figure 6B shows that electrical coupling among AII amacrine cells produces increases in the line-averaged dynamic range of the order of 4–7 percent in comparison with the uncoupled AII amacrine cells case.

## Discussion

In this work, we have used a minimal model of the primary and secondary ON rod processing pathways of the vertebrate retina to investigate the effects of some possible dynamic range-enhancing parameters, namely electrical coupling by gap junctions among rods and AII amacrine cells and maximal conductance 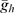 of rod I*_h_* current. The minimal model consisted of a single ganglion cell and the other retina cells of the ON pathways presynaptic to it arranged in a way that preserves the convergence and divergence factors along these pathways.

The main motivation for our study were some recent theoretical works [10]–[13], which suggest that electrical coupling among receptor cells in the periphery of a sensory system improves the dynamic range of the sensory system. Since in our model the populations of rods and AII amacrine cells are arranged in different grids, one receiving sensory inputs earlier than the other, we could investigate the effects of electrical coupling among these two cell populations independently of one another. Another motivation, which results from the fact that we used conductance-based model neurons in our work, was to study the combined effects of rod I*_h_* current [15], [16] with the electrical connectivity of rods and AII amacrine cells on the dynamic range of the retina.

Our results show that electrical coupling among rods and AII amacrine cells produces, as a general effect, an increase in the dynamic range of the vertebrate retina. This confirms the predictions of the theoretical works that motivated this work.

An important result is that the increase in dynamic range is much larger for the case of rod coupling than for the case of coupling among AII amacrine cells. This result is consistent with the idea that the greater part of the dynamic range enhancement along a sensory processing pathway should be implemented as peripherically as possible to avoid early saturation effects [10], [45]. Based on this result, we predict that selective blocking or damage to gap junction connections in the photoreceptors layer will have a much more important effect on the dynamic range of scotopic vision than selective blocking or damage to gap junction connections in the AII amacrine cells layer.

The results also show that the dynamic range-enhancing effects of either 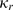 or 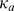 and rod 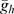, even when small, are not independent. These variables interact and cooperate so that only some specific combinations of them result in significant enhancements of the dynamic range of the vertebrate retina. Our results show that the dynamic range is maximized for a specific region of the 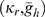 diagram. This region corresponds to a case of diluted rod-rod coupling combined with high values of rod 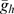.

This latter result may be due to the fact that for the 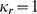 connectivity case the average number of activated rods is small. Hence, because of the high convergence factor from rods to rod bipolar cells and the saturation of the chemical synapse between a rod and a rod bipolar cell the rod bipolar cells are expected to have their average membrane voltage closer to saturation for the cases in which 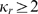 rather than in the cases where 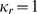. Besides, it is known that high values of the rod I*_h_* conductance reduce its voltage amplitude and prevent early saturation of its chemical synapse onto the rod bipolar cell [14], [15]. These two effects, one due to rod to rod coupling and the other due to an intracellular rod mechanism, interact with each other to enhance the dynamic range in our model. Based on this we predict that by differentially blocking the rod I*_h_* current one could experimentally manipulate the dynamic range of scotopic vision in the vertebrate retina.

The response curve of the ganglion cell for the range of inputs considered and for the parameter configuration which gave the largest dynamic range observed by us (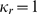, 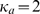 and 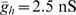) could be well approximated by a power law with exponent 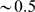. This is in direct agreement with predictions from models less detailed than ours [10]–[13]. The fact that approximately the same response behavior emerged from two very different models, a detailed model of the rod pathways in the retina like the one of this work and a simplified cellular automata model of a sensory epithelium like the ones of [10]–[13], suggests that they may be capturing the same basic dynamic range-amplification mechanism.

Moreover, our result may be related to the prediction from these models that sensory systems operate at or near a critical point of a phase transition. Recent evidence for power laws in the developing retina [40], cortical slices [41], [42] and functional brain networks obtained from fMRI and MEG data [43], [44] offer further support to the idea that neural systems attain optimal information processing capabilities at criticality. In our model, in particular, one could conjecture that the set of parameters that give maximum dynamic range and power law response function for the ganglion cell would correspond to a critical branching process [41] in the information transmission in the retina. However, it would be very hard to prove this conjecture with our model because of its three-dimensional structure and finite size.

### Conclusion and perspectives

The *in silico* investigation described in this work has shown that rod-rod coupling via gap junctions, in combination with appropriate values of the maximal rod I*_h_* current conductance, enhances the dynamic range of scotopic vision in the vertebrate retina. This confirms predictions from previous theoretical works [10]–[13]. Furthermore, it also has shown that the effect of gap junctional coupling among AII amacrine cells on the dynamic range is less pronounced than the one of rods coupling. This agrees with the intuitive notion that dynamic rance mechanisms should be implemented at the earliest possible processing stage [10], [45].

Our biologically detailed model of the rod pathways in the vertebrate retina gave results which are compatible with predictions from more abstract models. These predictions are consistent with experimental data [2], [3]. This illustrates, on the one hand, the usefulness of simple models for the understanding of brain function, and, on the other hand, that it is possible to have a “smooth” transition to models with a higher level of detail.

Further investigations are required in order to advance our understanding of the roles of cell connectivity and membrane properties on the dynamic range of the retina. We conclude that both simplified and realistic models, which respect the anatomy and physiology of the retina, will play a distinctive role in this endeavor.

## Supporting Information

Text S1 (0.07 MB PDF)

Table S1 Equations for the rod photoreceptor model.
(0.00 MB TEX)

Table S2 Equations for the cone photoreceptor model.
(0.00 MB TEX)

Table S3 Equations for the bipolar cell model.
(0.00 MB TEX)

Table S4 Equations for the AII amacrine cell model.
(0.00 MB TEX)

Table S5 Equations for the ganglion cell model.
(0.00 MB TXT)
