# Supplementary material for: A Computational Study on the Role of Gap Junctions and Rod Ih Conductance in the Enhancement of the Dynamic Range of the Retina
Source: PLoS One. 2009 Sep 24;4(9):e6970. doi: 10.1371/journal.pone.0006970 (PMC2745074; doi:10.1371/journal.pone.0006970)
Supplement: Text S1 — (0.07 MB PDF) [file pone.0006970.s001.pdf]

A computational study on the role of gap junctions and rod I_h conductance in the enhancement of the dynamic range of the retina

Rodrigo Publio[1*], Rodrigo F. Oliveira[2], Antonio C. Roque[3]

1 Computational Neuroscience Unit, Okinawa Institute of Science and Technology. 7542 Onna, Onna-son, Okinawa 904-0411, Japan.

2 Computational and Experimental Neuroplasticity Laboratory. The Krasnow Institute for Advanced Study,George Mason University, Fairfax,VA, USA

3 Department of Physics and Mathematics, FFCLRP. University of São Paulo, Ribeirão Preto, Brazil

∗ **E-mail: Corresponding publio@oist.jp**

Supporting Information

The retina conductance based model was created using the NEURON software [37] and it is composed of single compartment neuron models with passive parameters described in Table 1 of the main text. The conductances and reversal potentials for the neuron models are described in Table 1. For all conductances in the model we used the Hodgkin-Huxley formulation [46] where the activation, inactivation and non-inactivating variables (m,n,h) were numerically calculated as :

$$\frac{dx}{dt} = \alpha(1-x) - \beta x \tag{1}$$

where x represents any of the m,n,h variables, and α and β are the voltage-dependent activation and inactivation rates.

For all compartments in the neuron models that express calcium dependent channels the same calcium removal mechanism was used. The calcium influx is controlled by the voltage gated calcium and calcium dependent channels while the efflux is given by the second term in the following expression :

$$\frac{d[Ca^{+2}]_i}{dt} = \frac{-kI_{Ca}}{2Fd} - \frac{K_T[Ca^{+2}]_i}{[Ca^{+2}]_i + K_d} \tag{2}$$

where $k = 0.1$, $d = 1\mu m$, $K_T = 10^{-4}mM/ms$, $K_d = 10^{-4}mM$ and F is the Faraday constant.

Rod photoreceptor

The rod model is based on the single compartment model described in our previous work [16]. The modified model has a potassium current with fast dynamics responsible to accelerate the response to light stimulus (I_{Kx}), a potassium current with rectifier characteristics (I_{Kv}), a calcium dependent potassium current (I_{KCa}), a hyperpolarization activated current (I_h), a L-type calcium current (I_{Ca}) and a calcium dependent chloride current ($I_{Cl(Ca)}$) with parameters and dynamics taken from the references indicated in Table 2 and Table 4.

Cone photoreceptor

The cone model is a modified version of the single compartment model described by Kourennyi and others [17]. The modified model has a potassium current with rectifier characteristics (I_{Kv}), a calcium dependent potassium current (I_{KCa}), a hyperpolarization activated current (I_h), a calcium current (I_{Ca}) and a calcium dependent chloride current ($I_{Cl(Ca)}$) with parameters and dynamics taken from the references indicated in Table 2 and Table 4.

Rod and cone bipolar cell

The bipolar cells participate in both the rod and cone circuits connecting AII amacrine cells and ganglion cells [2, 3]. In this work we used the same model to simulate both the rod bipolar cell and the cone bipolar cell. The only difference between the single compartment models is that the rod bipolar cell model expresses the TEA sensitive I_A current [19]. The model is based on the single compartment model described by Usui and others [19] and has a potassium current with rectifier characteristics (I_{Kv}), a calcium dependent potassium current (I_{KCa}), a hyperpolarization activated current (I_h), a calcium current (I_{Ca}) and a TEA-sensitive potassium current (I_A) with parameters and dynamics taken from the references indicated in Table 2 and Table 6.

AII amacrine cell

The AII amacrine cell model is a modified version of the single-compartment model described by Smith and Vardi [24]. The modified model has only the Hodgkin-Huxley sodium and potassium channels [46] with parameters and dynamics taken from the references indicated in Table 2 of the main text and Table 7. The potassium current is activated when the cell is depolarized to -20mV while the sodium current is activated at -30 mV [24].

Ganglion cell

The ganglion cell model is a modified version of the single-compartment model described by Fohlmeister and Miller [25]. The modified model has a potassium current with rectifier characteristics (I_{Kv}), a calcium dependent potassium current (I_{KCa}), a calcium current (I_{Ca}), a TEA-sensitive potassium current (I_A) and the Hodgkin-Huxley sodium channels with parameters and dynamics taken from the references indicated in Table 2 and Table 8.

References

1. Sterling P, Freed MA, Smith RG (1988) Architecture of rod and cone circuits to the *on*-beta ganglion cell. J Neurosci 8: 623-642.

2. Deans MR, Volgyi B, Goodenough D, Bloomfield S, Paul D (2002) Connexin36 is essential for transmission of rod-mediated visual signals in the mammalian retina. Neuron 36: 703-712.

3. Volgyi B, Deans M, Paul D, Bloomfield L (2004) Convergence and segregation of the multiple rod pathways in mammalian retina. J Neurosci 24: 11182-11192.

4. Veruki ML, Oltedal L, Hartveit E (2008) Electrical Synapses Between AII Amacrine Cells: Dynamic Range and Functional Consequences of Variation in Junctional Conductance. J Neurophysiol 100: 3305-3322.

5. Veruki ML, Hartveit E (2009) Meclofenamic Acid Blocks Electrical Synapses of Retinal AII Amacrine and ON-Cone Bipolar Cells. J Neurophysiol 101: 2339-2347.

6. Tessier-Lavigne M, Attwell D (1988) The effect of photoreceptor coupling and synapse nonlinearity on signal:noise ratio in early visual processing. Proc R Soc Lond B 234: 171-197.

7. Hornstein EP, Verweij J, Li PH, Schnapf JL (2005) Gap-junctional coupling and absolute sensitivity of photoreceptors in macaque retina. J Neurosci 25: 11201-11209.

8. Vardi N, Smith R (1996) The aii amacrine network: coupling can increase correlated activity. Vision Res 36: 37433757.

9. Petrides A, Trexler EB (2008) Differential output of the high-sensitivity rod photoreceptor: Aii amacrine pathway. J Comp Neurol 507: 1653-1662.

10. Copelli M, Roque AC, Oliveira RF, Kinouchi O (2002) Physics of psychophysics: Stevens and weber-fechner laws are transfer functions of excitable media. Phys Rev E 65: 060901.

11. Copelli M, Oliveira RF, Roque AC, Kinouchi O (2005) Signal compression in the sensory periphery. Neurocomputing 63-64: 691-696.

12. Furtado LS, Copelli M (2006) Response of electrically coupled spiking neurons: A cellular automaton approach. Physical Review E 73:011907.

13. Kinouchi O, Copelli M (2006) Optimal dynamical range of excitable networks at criticality. Nature Physics 2: 348-351.

14. Baylor DA, Matthews G, Nunn BJ (1984) Location and function of voltage-sensitive conductances in retinal rods of salamander, *Ambystoma tigrinum*. J Physiol 354: 203-223.

15. Kamiyama Y, Ogura T, Usui S (1996) Ionic current model of the vertebrate rod photoreceptor. Vision Res 36: 4059-4068.

16. Publio R, Oliveira RF, Roque AC (2006) A realistic model of rod photoreceptor for use in a retina network model. Neurocomputing 69: 1020-1024.

17. Kourennyi D, Liu X, Hart J, Mahmud F, Baldridge W, et al. (2004) Reciprocal modulation of calcium dynamics at rod and cone photoreceptor synapses by nitric oxide. J Neurophysiol 92: 477-483.

18. Liu XD, Kourennyi DE (2004) Effects of tetraethylammonium on kx channels and simulated light response in rod photoreceptors. Ann Biomed Engineer 32: 1428-1442.

19. Usui S, Ishihara A, Kamiyama Y, Ishii H (1996) Ionic current model of bipolar cells in the lower vertebrate retina. Vision Res 36: 4069-4076.

20. Kaneko A, Pinto LH, Tachibana M (1989) Transient calcium current of retinal bipolar cells of the mouse. J Physiol 410: 613-629.

21. Yu-Ping M, Cui J, Hu H, Pan Z (2003) Mammalian retinal bipolar cells express inwardly rectifying k^+ currents (i_{Kir}) with a different distribution than that if i_h. J Neurophysiol 90: 34793489.

22. Berntson A, Smith R, Taylor W (2004) Postsynaptic calcium feedback between rods and rod bipolar cells in the mouse retina. Visual Neurosci 21: 913-924.

23. Boss R, Schneider H, Wassle H (1993) Voltage and transmitter gated currents of aii amacine cells in a slice preparation of the rat retina. J Neurosci 13: 2874-2888.

24. Smith RG, Vardi N (1995) Simulation of the aii amacrine cell of mammalian retina: Functional consequences of electrical coupling and regenerative membrane properties. Visual Neurosci 12: 851-860.

25. Fohlmeister J, Miller R (1997) Impulse encoding mechanisms of ganglion cells in the tiger salamander retina. J Neurophysiol 78: 1395-1947.

26. Zhang J, Wu SM (2005) Physiological properties of rod photoreceptor electrical coupling in the tiger salamander retina. J Physiol 564: 849-863.

27. von Gersdorff H (2001) Synaptic ribbons: versatile signal transducers. Neuron 29: 7-10.

28. Lagnado L (2003) Ribbon synapses. Curr Biol 13: R631.

29. Sikora MA, Gottesman J, Miller RF (2005) A computational model of the ribbon synapse. J Neurosci Methods 145: 47-61.

30. Mulloney B (2003) During fictive locomotion, graded synaptic currents drive bursts of impulses in swimmeret motor neurons. J Neurosci 23: 59535962.

31. Jacoby RA, Wu SM (2001) Ampa-preferring receptors mediate excitatory non-nmda responses of primate retinal ganglion cells. Vis Neurosci 18: 703-710.

32. Mukai S, Mishima HK, Shoge K, Shinya M, Ishihara K, et al. (2002) Existence of ionotropic glutamate receptor subtypes in cultured rat retinal ganglion cells obtained by the magnetic cell sorter method and inhibitory effects of 20-hydroxyecdysone, a neurosteroid, on the glutamate response. Jap J Pharmacol 89: 44-52.

33. Awatramani GB, Slaughter MM (2001) Intensity-dependent, rapid activation of presynaptic metabotropic glutamate receptors at a central synapse. J Neurosci 21: 741-749.

34. Rodieck RW (1988) The primate retina. Comp Primate Biol 41: 203-278.

35. LI W, DeVries S (2004) Separate blue and green cone networks in the mammalian retina. NatNeurosci 7: 745-750.

36. Pugh EN, Lamb TD (2000) Phototransduction in vertebrate rods and cones: molecular mechanisms of amplification, recovery and light adaptation. In: Preencher, editor, Handbook of Biological Physics, Amsterdam: Elsevier. pp. 183-255.

37. Hines ML, Carnevale NT (1997) The neuron simulation environment. Neural Comput 9: 1179-1209.

38. Carnevale T, Hines M (2006) The NEURON Book. Cambridge: Cambridge University Press, first edition.

39. Jones G (1995) Light adaptation and the rising phase of the flash photocurrent of salamander retinal rods. J Physiol 487: 3: 11167-11177.

40. Hennig MH, Adams C, Willshaw D, Sernagor E (2009) Early-Stage Waves in the Retinal Network Emerge Close to a Critical State Transition between Local and Global Functional Connectivity. J Neurosci 28: 1077-1086.

41. Beggs JM, Plenz D (2003) Neuronal avalanches in neocortical circuits. J Neurosci 487: 441-451.

42. Shew WL, Yang H, Petermann T, Roy R, Plenz D (2009) Neuronal avalanches imply maximum dynamic range in cortical networks at criticality. arXiv:0906.0527.

43. Eguluz VM, Chialvo DR, Cecchi GA, Baliki M, Apkarian AV (2005) Scale-free brain functional networks Phys Rev Lett 94: 018102.

44. Kitzbichler MG, Smith ML, Christensen SR, Bullmore E (2009) Broadband criticality of human brain network synchronization. PLoS Comput Biol 5: e1000314.

45. Stevens S (1975) Psychophysics: Introduction to its Perceptual, Neural and Social Prospects. New York: Wiley, first edition.

46. Hodgkin AL, Huxley AF (1952) A quantitative description of membrane current and its application to conduction and excitation in nerve. J Physiol 117: 500-544.

47. Ogura T, Sato TO, Usui S (2003) A simulation analysis on mechanisms of damped oscillation in retinal rod photoreceptor cells. Vision Res 43: 2019-2028.

48. Mao BQ, Maclish PR, Victor JD (2003) Role of Hyperpolarization-Activated Currents for the Intrinsic Dynamics of Isolated Retinal Neurons. Vision Res 83: 2756-2767.
